# Dissection of the mutation accumulation process during bacterial range expansions

**DOI:** 10.1186/s12864-020-6676-z

**Published:** 2020-03-23

**Authors:** Lars Bosshard, Stephan Peischl, Martin Ackermann, Laurent Excoffier

**Affiliations:** 1 CMPG, Institute of Ecology an Evolution, University of Berne, Baltzerstrasse 6, 3012 Berne, Switzerland; 2 Swiss Institute of Bioinformatics, 1015 Lausanne, Switzerland; 3 Interfaculty Bioinformatics Unit, University of Berne, 3012 Berne, Switzerland; 4 Institute of Biogeochemistry and Pollutant Dynamics, Swiss Federal Institute of Technology Zurich (ETH Zürich), 8092 Zürich, Switzerland; 5 Department of Environmental Microbiology, Swiss Federal Institute of Aquatic Science and Technology (Eawag), 8600 Dübendorf, Switzerland

**Keywords:** Experimental evolution, Range expansion, Mutation load

## Abstract

**Background:**

Recent experimental work has shown that the evolutionary dynamics of bacteria expanding across space can differ dramatically from what we expect under well-mixed conditions. During spatial expansion, deleterious mutations can accumulate due to inefficient selection on the expansion front, potentially interfering with and modifying adaptive evolutionary processes.

**Results:**

We used whole genome sequencing to follow the genomic evolution of 10 mutator *Escherichia coli* lines during 39 days ( ~ 1650 generations) of a spatial expansion, which allowed us to gain a temporal perspective on the interaction of adaptive and non-adaptive evolutionary processes during range expansions. We used elastic net regression to infer the positive or negative effects of mutations on colony growth. The colony size, measured after three day of growth, decreased at the end of the experiment in all 10 lines, and mutations accumulated at a nearly constant rate over the whole experiment. We find evidence that beneficial mutations accumulate primarily at an early stage of the experiment, leading to a non-linear change of colony size over time. Indeed, the rate of colony size expansion remains almost constant at the beginning of the experiment and then decreases after ~ 12 days of evolution. We also find that beneficial mutations are enriched in genes encoding transport proteins, and genes coding for the membrane structure, whereas deleterious mutations show no enrichment for any biological process.

**Conclusions:**

Our experiment shows that beneficial mutations target specific biological functions mostly involved in inter or extra membrane processes, whereas deleterious mutations are randomly distributed over the whole genome. It thus appears that the interaction between genetic drift and the availability or depletion of beneficial mutations determines the change in fitness of bacterial populations during range expansion.

## Background

Many populations expanded or shifted their range in their evolutionary history, for instance during the invasion of new habitats or in response to environmental changes [1–3]. Understanding the impact of dynamic species range margins on the evolutionary forces driving genomic and phenotypic evolution has become an important question in evolutionary biology, for example in the context of the evolution of dispersal [4], genetic diversity [5] or the structure of biodiversity [6]. Recent theoretical and empirical studies show that new mutations occurring at the edge of an expanding population can increase in frequency and spread over a large proportion of newly colonized territories. This process has been called gene surfing [7] and results from stochastic evolutionary processes at the wave front where population density is low and genetic drift is strong [8–10]. Theoretical studies have predicted that gene surfing should not only occur for neutral mutations, but also for mildly deleterious mutations. Deleterious mutations can thus accumulate during range expansion [11] and create an expansion load [12]. This prediction could be confirmed experimentally with expanding *Escherichia coli* populations [13].

Although the theory predicts that the fitness of spatially expanding populations of bacteria should decrease over time, there is evidence that populations that expand their range can evolve greater expansion speed [14–16], which can be a result of spatial sorting [4]. It remains unclear, however, if and how various evolutionary dynamics changes forces vary over time and space in populations that are expanding their range. Recently, microbial evolution experiments in liquid media using time-resolved sequencing have revealed complex dynamics occurring that are characterized by rapid adaptation, competition between beneficial mutations, epistasis, and genetic parallelism [17–20]. It is possible that adaptation is mainly due to constant selection occurring on mutations of small effect, which would lead to a gradual change in fitness. Alternatively, evolution on rugged fitness landscapes could lead to alternating periods of rapid phenotypic evolution and more static periods of evolution [21]. This variation in the rate of adaptation can be caused by changes in the environment, opportunities for improvement after key innovations, and invasion of new habitats [22, 23].

In this study, we investigate the rate at which mutations accumulate during range expansion by performing evolution experiments with populations of the bacterium *Escherichia coli*. We selected 12 populations from our previous experiment that expand their range on solid surfaces of agar plates for a total of 39 days [13]. We sequenced 6 samples at 13 time point and 6 samples at 5 time points within 39 days of expansion to determine for each line how many mutations accumulate over time. Additionally, we used the measurement of the expansion speed of the lines during the experiment to determine the effect of these mutations on the expansion speed and how these effects change over time.

## Results

### Linear increase in number of mutations and decrease of colony size over time

We sequenced the genome of 12 lines of *Escherichia coli* every third day for 39 days in total of radial expansion on agar plates. In total, we collected 108 DNA samples of the 12 lines during the 39 days of expansion (see Methods). Two lines were excluded after DNA sequence analysis due to contaminations during DNA extraction and/or library preparation. We thus used 90 sequences from 10 lines for all further analyses. The colony size was also measured after every growth period of 3 days.

**Fig. 1:**
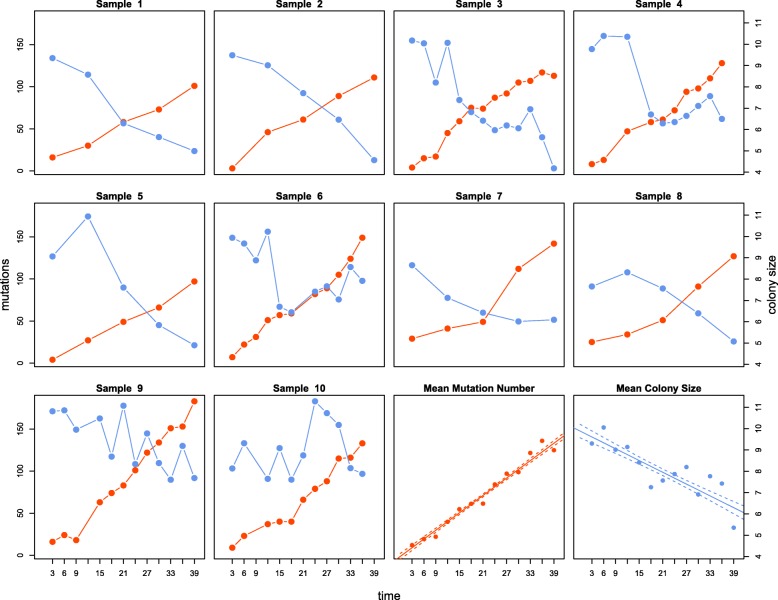
Dynamics of mutation accumulation and colony size over time for samples 1–10. Blue: Change in bacterial colony radius measured after three days of expansion on agar plates. Red: Number of mutations accumulated in bacterial lines over the 39 days of expansion. The last two panes display the mean number of mutations and the mean colony sizes computed over the 10 samples for each time period. Solid lines indicate regression lines and dashed lines delimit 95% confidence intervals

We used a linear mixed effect regression model to predict expansion speed over time, and, separately, the number of accumulated mutations. In the first mixed effect model used to predict expansion speed, we estimated an individual random effect for the intercept and the slope of the linear model (to account for the dependence of the measurements over time for each line). On average, colony size, measured as the radius at the end of a 3-day expansion period, decreased at a rate of 95 μm per day (95% CI: [− 129,-62]; *p*-value < 2.2 × 10^− 16^) over the course of the experiment (Fig. 1). The lines accumulated on average 3.1 mutations per day (95% CI: [2.45, 3.71], Fig. 1). For the colony size data, the linear model explains about 67% of the variation (R_c_^2^ = 0.67) indicating that there is still considerable variation that this simple model cannot explain. This is not surprising since there are several unaccounted factors that potentially have an impact colony size, i.e. variation of mutation effect size, temperature, humidity, agar concentration, and fluctuations in nutrition composition. In contrast, the model used to predict number of mutations explains 95% of variation in the data (R_m_^2^ = 0.95) suggesting that mutations accumulate almost linearly over time. The linear accumulation of mutations suggests that the mutation rate and the generation time remained largely constant over the course of the experiment and shows that evolutionary changes in colony size did not impact the rate at which mutations accumulate. If the colony size data are split in four periods and the mixed effect model is used to analyze the time periods separately, the slope is not significantly different from 0 at period 3–12 days (*p* = 0.5391), 21–30 days (*p* = 0.4352), and 30–39 days (*p* = 0.0529) (Supplementary Figure 2). However, there is a significantly negative slope in the period 12–21 days (*p* = 0.0142), suggesting that the colony size only decreases significantly in the second period (day 12–21) and that it does not change significantly in the other periods. (Supplementary Figure 2).

### dN/dS ratio decreases over time

**Fig. 2:**
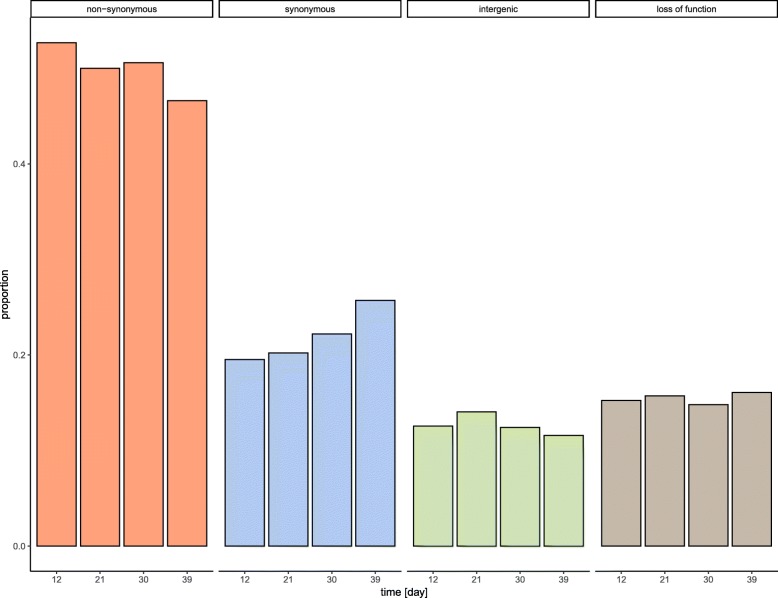
Change in mutation types over time: Bar plot of the proportion of different mutations over time. Orange: non-synonymous mutations, blue: synonymous mutations, green: loss of function mutations, brown: intergenic mutations

We analyzed the mutations in four consecutive time periods: Mutations that occurred in days 3–12, days 12–21, days 21–30, and days 30–39, respectively (Fig. 2). The analysis of the dN/dS ratio change over time suggests that there is a larger proportion of non-synonymous mutations than synonymous mutations at the beginning of the experiment (dN/dS = 1.4754, *p* = 0.0041) (Fig. 2, and Table 1) indicative of positive selection during this early phase. The dN/dS ratio is not significantly different from 1 in the later period of the evolution experiment (Table 1) indicating that non-synonymous and synonymous mutations accumulate randomly at later stages. The dN/dS ratio is significantly different between day 3–12 and day 30–39 (*p* = 0.039). All other pairwise comparisons between the different time periods are not significant.

**Table 1:** dNdS ratio calculated for mutations occurring in four time periods (3–12, 12–21, 21–30, and 30–39 days). Reported *p*-values were obtained by a permutation test

	day 3–12	day 12–21	day 21–30	day 30–39
dN/dS	1.4754	1.3516	1.2463	0.9909
*p* value	0.0041	0.1348	0.0823	0.9445

### The effects of mutations on colony size shifts become more negative over time

We used an Elastic Net (EN) regression, which performs both variable selection and variable regularization, to determine the subset of genes that have the largest effect on colony size by analyzing non-synonymous and loss of function (LOF) mutations. This analysis estimates for each gene the effect a mutation has on colony size. Positive values indicate that a mutation causes an increase in colony size and negative values indicate a decrease in colony size. We used the change in colony size between two sampling points and a list of genes with new mutations during the two sampling points for the EN analysis. There were 6 genes remaining in the model associated with an increased colony size and 34 genes associated with a colony size reduction (Table 2). 15 genes out of the 34 genes are involved in metabolic processes, 15 genes are connected to the formation of cell membrane, transporter proteins, and motility, and 5 genes are controlling gene expression and DNA structure.

**Table 2:** Effects of non-synonymous and loss of function mutations on colony size, as inferred by Elastic Net regression. Effect sizes are relative to the initial colony size. The functional units were defined using Ecocyc [24]

• Name	• Gene description	• Pos. Coef.	• Neg. Coef.	• Function unit
• croE	• RNA polymerase assembly factor	• 0.867	•	• DNA or RNA process
• livM	• Transporter	• 0.705	•	• Transporter
• ybiO	• Transporter	• 0.243	•	• Transporter
• ycfQ	• Transcriptional repressor	• 0.679	•	• Regulator
• fdoG	• Formate dehydrogenase	• 0.627	•	• Metabolic process
• ybdH	• Swarming motility	• 0.066	•	• Motility
• yheT	• Predicted hydrolase	•	• −3.766	• Metabolic process
• frlD	• Phosphorylation	•	• −0.695	• Metabolic process
• metL	• Amino acid biosynthesis	•	• −0.686	• Metabolic process
• pdxJ	• Metabolic process	•	• −0.596	• Metabolic process
• fixC	• Flavoprotein	•	• −0.593	• Metabolic process
• glnE	• Glutamine synthesis	•	• −0.533	• Metabolic process
• yphB	• Conserved protein	•	• −0.508	• Metabolic process
• yfeS	• Conserved protein	•	• −0.381	• Metabolic process
• ybhJ	• Metabolic process	•	• −0.181	• Metabolic process
• elbB	• Lycopene biosynthesis	•	• −0.177	• Metabolic process
• panC	• Biosynthetic process	•	• −0.104	• Metabolic process
• msyB	• Heat sensitivity	•	• −0.081	• Metabolic process
• gtrB	• Prophage	•	• −0.076	• Metabolic process
• hpc	• Nitrate metabolism	•	• −0.044	• Metabolic process
• dmlA	• D-malate dehydrogenase	•	• −0.032	• Metabolic process
• yfiL	• Lipoprotein	•	• −1.484	• Membrane
• wcaL	• Colanic acid synthesis	•	• −0.507	• Membrane
• lnt	• Lipoprotein	•	• −0.228	• Membrane
• yfjD	• Inner membrane protein	•	• −0.124	• Membrane
• yciM	• Lipopolysaccharide assembly	•	• −0.072	• Membrane
• ddpA	• Peptide ABC transporter	•	• −0.904	• Transporter
• fecC	• Transporter	•	• −0.751	• Transporter
• yqcE	• Transporter	•	• −0.282	• Transporter
• pheP	• Phenylalanine transporter	•	• −0.103	• Transporter
• alsA	• Transporter	•	• −0.081	• Transporter
• ccmB	• Transporter	•	• −0.073	• Transporter
• uidB	• Glucuronide transporter	•	• −0.045	• Transporter
• paaX	• Regulator	•	• −0.784	• Regulator
• rssB	• Regulator of RpoS	•	• −0.649	• Regulator
• preA	• Swarming motility	•	• −0.497	• Motility
• yeaJ	• Motility	•	• −0.011	• Motility
• recG	• DNA repair	•	• −0.245	• DNA or RNA process
• der	• Ribosomal stability factor	•	• −0.238	• DNA or RNA process
• leuP	• tRNA	•	• −0.188	• DNA or RNA process

**Fig. 3:**
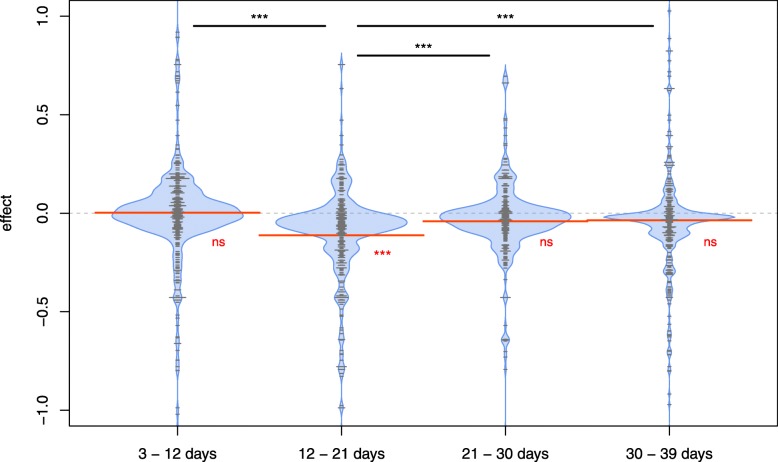
Mutation effect dynamics: Distribution of mutation effects over colony growth. The mutations are distributed into four time periods. Horizontal grey lines represent mutations in a given gene and the length of the grey line is proportional to the number of mutations that were observed in that time period. Red lines indicate the mean value and red asterisks indicate if the mean value is significantly different from 0. 3–12 days: *p* = 0.7858; days 12–21: *p* < 2.2 10^− 16^; 21–30 days: *p* = 0.0627; 30–39 days: *p* = 0.1125. Black bars on top indicate if mutation mean effects in different time periods are significantly different from each other, based on a pairwise t test with Bonferroni correction for multiple testing: 3–12 days - days 12–21: *p* = 6.5 10^− 11^; days 12–21 - 21-30 days: *p* = 6.2 10^− 4^; days 12–21 - 30-39 days: *p* = 1.4 10^− 4^. All other pairwise comparisons are not significant

We additionally estimated mutation effects on colony growth in the four time periods (A: 3–12 days, B: 12–21 days, C: 21–30 days, and D: 30–39 days) by analyzing non-synonymous and loss of function (LOF) mutations with ridge regression, which performs only variable regularization without variable selection (Fig. 3). We estimated an effect for each gene, and took it into account even if it was close to zero. Therefore, we could investigate the distribution of the effects of all genes. The estimated mean mutation effect does not significantly from 0 in the first 12 days and after day 21 (3–12 days: *p* = 0.7858; 21–30 days: *p* = 0.0627; 30–39 days: *p* = 0.1125). Contrastingly, between days 12–21, we observe a significantly negative mean effect of a new mutation (*p* < 2.2 10–16) (Fig. 3). This result implies that there is either a shift to more deleterious mutations in the second period or that there are more beneficial mutations at the beginning of the experiment. The latter explanation is in line with the observed dN/dS ratio that is significantly larger than 1 during the first period.

### GO enrichment analysis

We investigated if there was a significant enrichment of non-synonymous and LOF mutations found to have an effect on colony size by our EN method (see Table 2) in gene ontology terms, and this for the four different time periods considered above as well as over the whole experiment. For this analysis, we used all genes irrespective of whether they had been affected by positive or negative mutations, since there were not enough mutations in each of these separate categories. We found two significantly enriched GO term using data from the entire experiment: organelle inner membrane (GO:0019866; q = 0.00017) and peptidoglycan-based cell wall (GO:0009274; q = 0.00202) (Supplementary Figure 1). Note that bacteria do not possess organelles, but genes in this GO term are defined as membrane-bounded structures with a specified protein content and specified biochemical output [25]. We find the same two significant GO terms in the first period (day 3–12): organelle inner membrane (GO:0019866; q = 0.01725) and peptidoglycan-based cell wall (GO:0009274; q = 0.01725). There were no significant GO terms after 12 days until the end of the experiment. The genes that are mutated in the two GO terms (GO:0019866, GO:0009274) can be further divided in four functional groups using Ecocyc [24]: flagella assembly, transporter and signaling proteins at the inner membrane, and peptidoglycan assembly of the cell wall (Supplementary Figure 1).

## Discussion

We investigated here the accumulation of mutations in 10 *Escherichia coli* lines over 39 days of expansion on agar plates. We analyzed the temporal dynamics of the effect of mutations on the speed of expansion of bacterial colonies on an agar plate. The focus was to identify the temporal dynamics of the interactions between selection and genetic drift during range expansions. We do not find here evidence of a constant decrease in fitness over time. Rather, the dynamics of fitness change is more complex, with the occurrence of a mixture of positively and negatively selected mutations at all stages, even though their relative proportions and effects vary over time (Figs. 2 and 3). Previous studies have shown that expansion speed could be also influenced by interactions among differentiated pioneering cells at the front of the expanding population [26]. However, in this study the standing variation in the ancestral population is expected to be low, and interactions between different cell types is therefore potentially limited.

We find evidence of positive selection driven by non-synonymous mutations in the first 12 days, as attested by a significant dN/dS ratio (dN/dS = 1.48, *p* = 0.0041, Table 1). However, the estimated average effect of non-synonymous and LOF mutations on colony size is not significantly different from 0 in the first quarter of the experiment (Fig. 3). It suggests that there are beneficial mutations in the first 12 days of the experiment that are compensating for the effect of other deleterious mutations, resulting in a null effect on fitness. There is then a significant decrease in fitness between days 12 and 21, but the dN/dS ratio is not deviating significantly from 1. The observation of a constant fitness at the beginning of the experiment and of a decreasing fitness at a later stage of the experiment could be due to a limited number of mutations that can lead to an increase in colony size [27]. After the reservoir of potential positive mutations is exhausted or becomes too small, we would indeed mainly see the effect of a constant accumulation of deleterious mutations, leading to a progressive decrease in the fitness of the bacteria on the front. Note that the rate of fitness gain declines also in well mixed (liquid growing) bacterial populations over time [28], but in contrast to an expanding populations on a two-dimensional surface, its molecular evolution is characterized by signatures of rapid adaptation during the experiment [28]. After 21 days, the mutational effects are not significantly different from 0 (Fig. 3), which is in line with the predictions of a Fisher Geometric Model where the proportion of beneficial mutations increases when a population gets further away from its optimum [29]. Under this line of reasoning, the accumulation of deleterious mutations during days 12 to 21 would have moved the lines away from their optimum, therefore allowing for a higher influx of beneficial mutations after 21 days. However, the effect is either not strong enough to see a significant dN/dS ratio after 21 days in our experiment, or it is mainly driven by LOF mutations.

In this study, we focused on the average effect of mutations among all 10 lines, but mutations occurring in an individual line can show a large deviation from this average effect. There is indeed quite a high variability in the fitness trajectories among different lines (Fig. 1), as the fitness of some lines continues to decrease after 21 days. The fact that the mean effect of the mutations is not significantly different from zero after day 21 on Fig. 3 is also potentially due our limited sample size. A larger study performed over a longer time period would be useful to draw more definitive conclusions. The fact that the number of mutations per line increases linearly over time suggests that mutations occur at a constant rate, which is in line with previous studies of *Escherichia coli* lines in liquid medium [28, 30], where the rate of genomic evolution was nearly constant. However, in the previous evolution experiments in liquid culture, the dN/dS ratio was significantly larger than one [30] and fitness increased after a short time period relative to the ancestor [19, 28, 31]. Our observation that the fitness decreases in the second period of the experiment (day 12–21) is in line with the theoretical predictions that natural selection is inefficient during range expansions due to low effective population size at the expanding front, leading to an inefficient purging of deleterious mutations [12, 32]. Expansion speed depends generally on dispersal and growth rate, but mutations can have a different impact on these two mechanisms, and these two traits tend to interact and co-evolve [11]. Interestingly, an increase in colony size has been predicted for expanding motile bacteria where faster dispersal can evolve [16]. Therefore, the relative strength of drift and selection might change over time [33].

The GO enrichment analysis performed on non-synonymous and LOF mutations revealed two significant GO terms in the total data set as well as in the first 12 days of evolution: organelle inner membrane (GO:0019866) and peptidoglycan-based cell wall (GO:0009274). The mutated genes belonging to these GO terms are coding for proteins functionally connected to the cell membrane and potentially involved in the surface structure of the cell ((Supplementary Figure 1). There is evidence that structural changes of surface proteins can lead to bacterial cell sorting, such as to more easily allow them to move to the front of the expansion by reducing drag [34]. Changes on the cell surface also potentially have an impact on the stability of the edge of the colony [35, 36]. By weakening the stability of the colony, the same number of bacteria could spread over a larger area, and lead to a thinner colony [15], since they would be less densely packed. Our results thus strongly suggest that some non-synonymous mutations in membrane protein genes occurring early during the experiment lead to an increase in colony size and are therefore positively selected. Previous estimates of the distribution of fitness effects (DFEs) over the whole experiment suggest that there are on average more deleterious mutations accumulating in during a long period of range expansion on agar plates [13], but the DFE results suggested that there were also many potentially positively selected mutations occurring during these expansions, even though it was not possible to individualize them. Due to the relatively small sample size (10 lines) and the smaller number of mutations observed in each time period, it was not possible to infer period specific DFEs, but we nevertheless show that these beneficial mutations accumulated early during the experiment. The study of a much larger number of strains could certainly enable one to examine if and how DFEs change over the course of the experiment.

## Conclusions

Our results highlight the importance of considering the spatially explicit process of bacterial growth when studying bacterial adaptation and evolution, as functional constraints imposed by range expansions could seriously limit the ability of bacteria to cope with environmental changes [37]. Complex adaptive processes demonstrated here in bacteria could also happen during the expansion of other populations, including humans, but also during the growth of solid tissues in eukaryotes. The analogy between the evolution of bacterial communities and the growth of eukaryotic tissue has recently been highlighted, in particular in cancer [38]. Like bacteria, solid cancers evolve by a process of clonal expansion, exploring the adaptive landscapes of tissue ecosystems [39]. Expansion load theory in non-recombining organisms could therefore also explain phenomena such as spontaneous tumor recession, irregular growth patterns, or extremely high clonal diversity in tumors [40–42]. In addition to having triggered the development of specific life-history traits in most organism (reviewed in [43]), the negative impact of deleterious mutations could have led to the development of specific cellular mechanism preventing their specific accumulation during tissue growth, and apoptosis could be such an example.

## Methods

### Bacterial strain

We used *Escherichia coli* K12 MG 1655 strains where the expression of the *mutS* gene is directly controlled by the arabinose promoter *pBAD* inserted in front of the *mutS* gene. In absence of arabinose, *mutS* is not expressed, leading to a higher spontaneous mutation rate due to the inactivation of the methyl-directed mismatch repair system (MMR, [44]). Additionally, our strain had a GFP marker located in the lac operon, which can be induced by IPTG (Isopropyl β-D-1-thiogalactopyranoside).

### Experimental setup

**Fig. 4:**
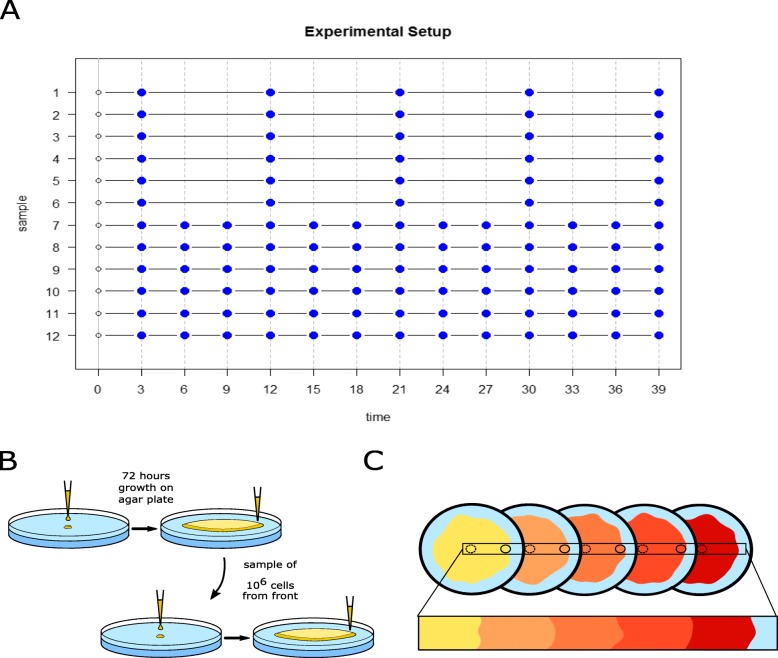
Sketch of the experimental setup. **a** Sampling design of the 12 bacterial lines evolved over the 39 days of the experiment. The vertical dashed grey line represents the transfer of the lines to a new agar plate, which occurred every three days. Blue dots indicate that the DNA of this line was extracted and sequenced at this time. **b** We transferred about one million cells taken from a random point on the edge of the colony after three days of growth to the center of a new agar plate. **c** The periodic transfer occurring every three days without any strong bottleneck aims at mimicking a continuous expansion in space

Twelve bacterial strains were propagated on LB agar plates at 37 °C for a total duration of 39 days. The strains were transferred on new agar plates every 3 days (Fig. 4). An image of the colony was taken before transferring the strains to a new plate. The location of the sampling point of each transfer was chosen at random on the periphery of the colony. At each transfer, a sample containing about 100 million cells was collected from the colony front using a sterile pipette tip and resuspended in 100 μl 0.85% NaCl solution. About one million cells were then used to inoculate a new plate (Fig. 4b). This expansion experiment on several plates aims at mimicking a continuous expansion for 39 day or 1650 generations (Fig. 4c). We extracted DNA from six lines during each of the 13 transfers, and for six other lines, we extracted DNA at day 3, 12, 21, 30, and 39. We thus analysed a total of 108 DNA samples from the 12 lines (Fig. 4a).

### DNA extraction

After the range expansion experiment on agar, one million cells from the wave front were streaked out on an LB agar plate containing 0.5% arabinose and incubated for 24 h at 37 °C to isolate single clones. A single colony was dissolved in 100 μl dilution solution (0.85% NaCl) and 1 μl was transferred to a new LB agar plate containing 0.5% arabinose. The plate was then incubated for 24 h at 37 °C. Then, the entire colony was removed from the agar plate and resuspended in 1 ml dilution solution. Genomic DNA was extracted using the Wizard Genomic DNA Purification Kit (Promega) following the manufacturer protocol. The integrity of the DNA was checked by gel electrophoresis. The DNA concentration was determined by fluorometric quantification (Qubit 2.0).

### Whole genome sequencing and variant calling

108 DNA samples of 12 lines were sequenced using a TruSeq DNA PCR-Free library (Illumina) on a HiSeq 3000 platform (Illumina), from which we obtained 100 bp paired end reads for all samples. Trimmomatic 0.32 [45] was used to remove the adapter sequences from the reads and for quality trimming. Leading and trailing bases with quality below 3 were removed. The reads were scanned with a 4 bp sliding window and cut if the average quality per base was below 15. Reads with a length below 36 were excluded from the analysis. Variants were identified using BRESEQ (version 0.27.2), a computational tool for analyzing short-read DNA data [46]. BRESEQ uses Bowtie2 (Langmead, et al. 2009) to map reads to the *Escherichia coli* K12 MG1655 (NC_000913.3) reference genome. As a first step, it identifies potential new junctions between disjoint regions of the reference sequence using all available reads. BRESEQ then uses an empirical error model for base quality re-calibration considering the identity of the reference base, the identity of the mismatch base, the base position within the read, and the neighboring base identities. At each alignment position, BRESEQ calculates the posterior probability of a given nucleotide given the observed aligned reads. If the nucleotide with the highest posterior probability is different from the reference, BRESEQ records read alignment evidence. The top/bottom strand distribution of reads supporting the major base is compared to the top/bottom distribution of reads supporting the minor base by using a Fisher’s Exact Test to avoid false-positive polymorphism prediction due to sequencing-error hotspots in reads on one strand. A one-sided Kolmogorov-Smirnov test was used to test whether base quality scores supporting the minor mutational variants are suspiciously lower than the base quality scores supporting the major variant. We excluded two sample after analyzing the DNA sequences due to potential contaminations.

### Estimation of dN/dS ratio

The number of synonymous and non-synonymous substitutions were computed in each line. The dN/dS ratio was then estimated by taking the expected number of synonymous and non-synonymous substitutions into account if all codon positions in the reference genome would have mutated. We used a bootstrap approach to test if the dN/dS ratio is significantly different from 1. dN/dS was computed using randomized data sets in which the mutations were randomly sampled with repetition among six types of non-synonymous and six types of synonymous mutations (four possible transition and two possible transversions).

### Analysis of colony size and number of mutations

We determined for each time point (Fig. 4a) the number of mutations that have accumulated in each of the 12 lines, as well as the corresponding colony size. After exclusion of one line due to contaminations we were left with 103 measurements of 11 lines between 3 and 39 days. We determined the change in colony size and the change in the number of mutations over time by fitting a mixed-effect linear model to the data. We fit a fixed effect slope ***β*** to the data that describes the effects common to all lines, and the model also considers line-specific variability in the slope by including random effects ***b***_*i*_ for the intercept and slope for the *i*-th line:

$$ {\boldsymbol{y}}_i={\boldsymbol{X}}_i\boldsymbol{\beta} +{\boldsymbol{Z}}_{\boldsymbol{i}}{\boldsymbol{b}}_i+{\boldsymbol{\varepsilon}}_i $$

$$ {\boldsymbol{b}}_i\sim N\left(\mathbf{0},\boldsymbol{\varPsi} \right) $$

$$ {\boldsymbol{\varepsilon}}_i\sim N\left(\mathbf{0},{\sigma}^2\boldsymbol{I}\right) $$

$$ i=1,\cdots, 20 $$

where ***X***_*i*_ and ***Z***_*i*_ are known fixed effect and random effect regressor matrices, ***ε***_*i*_ is the within group error with a spherical Gaussian distribution, and ***Ψ*** is the variance-covariance matrix of the random effects.

Two types of determination coefficients (R^2^) can be calculated for mixed effect regression models. The marginal ${R}_m^2$ represent the variance explained by the fixed effects of the model, whereas the conditional ${R}_c^2$ represents the variance explained by the entire model (with both fixed and random effects). The *r.squaredGLMM* function of the R package *MuMIn* was used to calculate ${R}_m^2$ and ${R}_c^2$. In the model for the change in colony size over time and the model for the change in number of mutations the addition of a random effect of the slope significantly improves the fit of the models compared to a model with only a random effect for the intercept (Likelihood ratio tests; for the colony size model: *p*-value = 0.0017; for the number of mutation model: p-value = < 0.0001).

### Effect of mutations on the colony size

The difference in colony size (**Δc**) arising from two consecutive expansions on agar plates, each of these expansions lasting for three days, was calculated for all lines and the mutations that accumulated during this period were determined. Only non-synonymous, frameshift, and non-sense mutations were considered, and for each **Δc**, the number of mutations (***M***) in every gene was determined. ***M*** has the same number of rows as the change of colony size **Δc** and 888 columns, one for every gene that had at least one mutation during the experiment. We used a regression approach to model the change in colony size **Δc** with the number of mutations in the genes ***M***:

$$ \boldsymbol{\Delta} \mathbf{c}=\boldsymbol{M}+\boldsymbol{\upvarepsilon} $$

where ε is the vector of residuals.

To avoid overfitting due to the high dimensionality of ***M***, ridge regression was used to estimate the effect of a mutation on colony size in a given gene. If a mutation in a gene has no effect on the colony size, ridge regression shrinks the coefficient close to zero. Positive coefficients indicate an increase of colony size and negative coefficients indicate a decrease. The shrinking of the parameters is controlled by the regularization parameter λ, whose value was chosen by 3-fold cross-validation using the *cv.glmnet* function of the *glmnet* package.

$$ {\beta}^{ridge}={argmin}_{\beta}\left(\sum \limits_{i=1}^N{\left({y}_i-{\beta}_0-\sum \limits_{j=1}^p{x}_{ij}{\beta}_j\right)}^2+\lambda \sum \limits_{j=1}^p{\beta}_j^2\right) $$

### Gene ontology enrichment test

We tested if there was a signal of adaptation during different periods of the experiment by using a gene ontology (GO) enrichment analysis where we only used non-synonymous, frameshift and nonsense mutations in each time period. The test was performed with the topGO package for R [47] on the genes that were detected to have a positive coefficient in the ridge regression. The resulting list of genes was used separately to perform a Fisher’s exact test to determine significantly over-represented GO terms. The *weight01* algorithms used in the topGo analysis iteratively removes the genes mapped to significant GO terms from higher level GO terms and the significance score of connected nodes are compared to detect the locally most significant terms in the GO graph by down-weighting genes in less significant neighbors. The GO enrichment was applied separately to the following time periods: days 3–12, days 12–21, days 21–30, and days 30–39.

## Supplementary information

**Additional file 1: Supplementary Figure 1**: Cellular location of the four main functional groups present in the two GO terms that are significant when using the data from the entire experiment and from days 3–12 (organelle inner membrane, GO:0019866; q = 0.01725; peptidoglycan-based cell wall, GO:0009274; q = 0.01725). There were no significant GO terms in the other time periods (12–21, 21–30, 30–39 days). **Supplementary Figure 2:** Colony size data split into four periods (3–12 days, 12–21 days, 21–30 days, and 30–39 days). The regression line inferred under a mixed effect model for each time period is shown in blue. The slope of the regression lien is not significantly different from 0 in periods 3–12 days (*p* = 0.5391), 21–30 days (*p* = 0.4352), and 30–39 days (*p* = 0.0529), but it is significantly negative in the period 12–21 days (*p* = 0.0142).

## Data Availability

The datasets generated and analysed during the current study are available in the Sequence Read Archive (SRA) repository, SRA accession: PRJNA611679.

## Abbreviations

EN: Elastic Net
LOF: Loss of Function
GO: Gene Ontology
DFEs: Distribution of Fitness Effects
GFP: Green Fluorescent Protein
IPTG: Isopropyl β-D-1-thiogalactopyranoside
MMR: Methyl-directed Mismatch Repair
LB: Lysogeny Broth
DNA: Deoxyribonucleic Acid
PCR: Polymerase Chain Reaction
