# Supplementary material for: Dissection of the mutation accumulation process during bacterial range expansions
Source: BMC Genomics. 2020 Mar 23;21:253. doi: 10.1186/s12864-020-6676-z (PMC7092555; doi:10.1186/s12864-020-6676-z)
Supplement: Supplementary file 1 — Additional file 1: Supplementary Figure 1: Cellular location of the four main functional groups present in the two GO terms that are significant when using the data from the entire experiment and from days 3–12 (organelle inner membrane, GO:0019866; q = 0.01725; peptidoglycan-based cell wall, GO:0009274; q = 0.01725). There were no significant GO terms in the other time periods (12–21, 21–30, 30–39 days). Supplementary Figure 2: Colony size data split into four periods (3–12 days, 12–21 days, 21–30 days, and 30–39 days). The regression line inferred under a mixed effect model for each time period is shown in blue. The slope of the regression lien is not significantly different from 0 in periods 3–12 days (p = 0.5391), 21–30 days (p = 0.4352), and 30–39 days (p = 0.0529), but it is significantly negative in the period 12–21 days (p = 0.0142). [file 12864_2020_6676_MOESM1_ESM.docx]

Supplementary Information


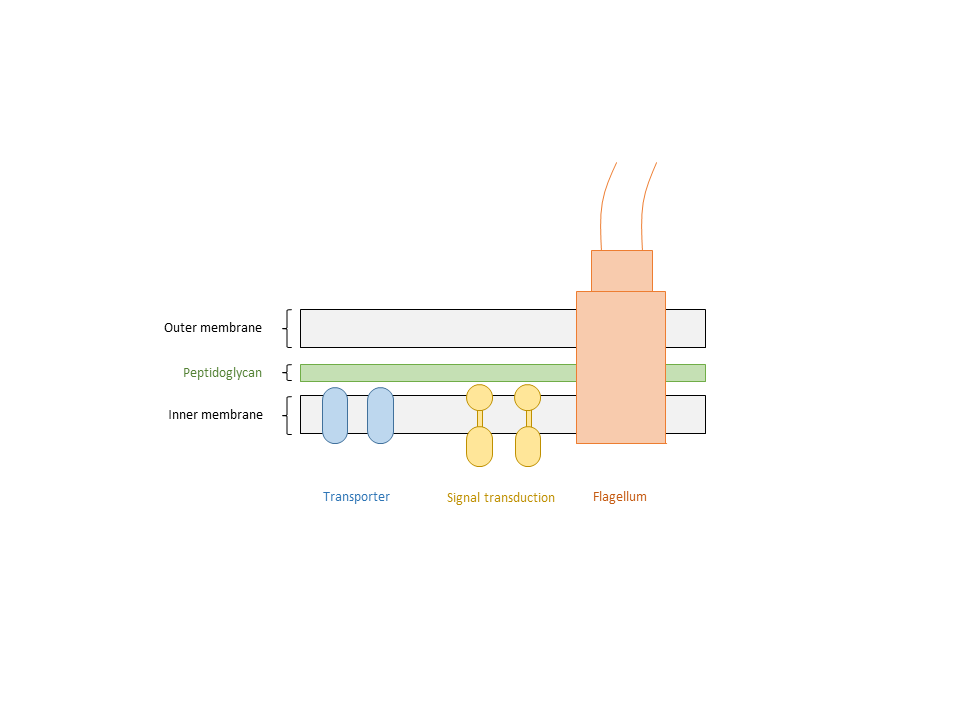


**Supplementary** **Figure 1**: Cellular location of the four main functional groups present in the two GO terms that are significant when using the data from the entire experiment and from days 3-12 (organelle inner membrane, GO:0019866; q = 0.01725; peptidoglycan-based cell wall, GO:0009274; q = 0.01725). There were no significant GO terms in the other time periods (12-21, 21-30, 30-39 days).


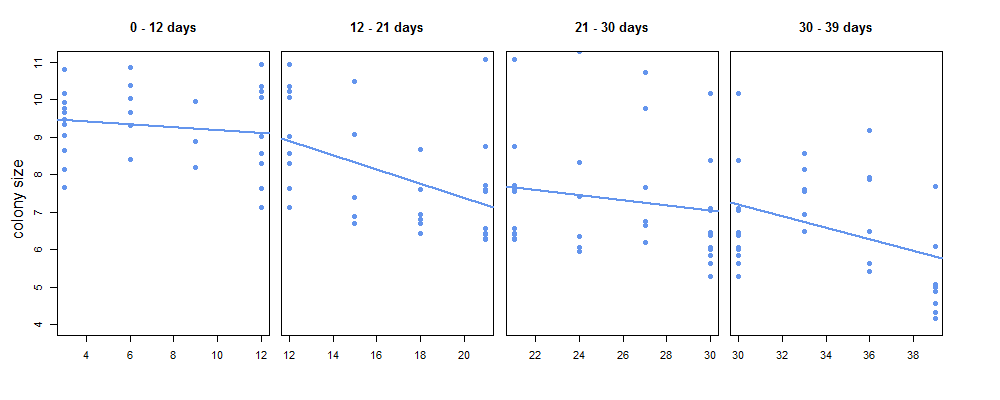


**Supplementary Figure 2:** Colony size data split into four periods (3-12 days, 12-21 days, 21-30 days, and 30-39 days). The regression line inferred under a mixed effect model for each time period is shown in blue. The slope of the regression lien is not significantly different from 0 in periods 3-12 days (p=0.5391), 21-30 days (p=0.4352), and 30-39 days (p=0.0529), but it is significantly negative in the period 12-21 days (p=0.0142).
